# Humanized APOE mouse brain volume increases over age irrespective of sex and APOE genotype: implications for translational validity to the human

**DOI:** 10.3389/fnins.2026.1843319

**Published:** 2026-07-14

**Authors:** Adam C. Raikes, Avnish Bhattrai, Tian Wang, Jean-Paul Wiegand, Roberta Diaz Brinton

**Affiliations:** 1 Center for Innovation in Brain Science, University of Arizona, Tucson, AZ, United States; 2 Department of Neurology, University of Arizona, Tucson, AZ, United States; 3 Department of Pharmacology, College of Medicine Tucson, University of Arizona, Tucson, AZ, United States

**Keywords:** aging, Alzheimer’s disease, APOE, brain volume, mouse model, MRI

## Abstract

**Background:**

Humanized APOE mouse models are widely used to study late-onset Alzheimer’s disease (LOAD) risk, yet it remains unclear whether they reproduce the macrostructural brain changes observed in human aging and disease.

**Methods:**

We performed *ex vivo* magnetic resonance imaging to quantify total and voxelwise brain volumes in male and female mice across APOE genotypes (ε3/ε3, ε3/ε4, ε4/ε4) and ages spanning 6–25 months.

**Results:**

Total brain volume increased with age (cross-sectional estimate: 2.12 mm^3^/month) and was greater in APOE-ε4 carriers, with no effect of sex. Voxelwise analyses revealed regionally specific changes independent of total volume, characterized by cortical volume decreases and subcortical preservation or increases, as well as sex-dependent spatial patterns. No localized volumetric effects of APOE genotype were detected.

**Conclusion:**

These findings indicate that, despite incorporating a major genetic risk factor for LOAD, this model does not reproduce the atrophy phenotype characteristic of human aging and Alzheimer’s disease. Instead, the observed pattern is more consistent with non-pathological or vulnerable aging, suggesting that humanized APOE alone is insufficient to induce MRI-detectable macrostructural atrophy within this cross-sectional comparison, though this finding does not preclude other APOE-dependent pathological mechanisms.

## 1 Introduction

Approximately 1 in 9 Americans over the age of 65 lives with late-onset Alzheimer’s disease (LOAD) (Alzheimers Dementia, 2025). Without effective treatments, the prevalence is expected to exceed 12.7 million by 2050 with new diagnoses exceeding 1 million per year (Alzheimers Dementia, 2025; CDC, 2025; Fang et al., 2025). The strongest risk factors for LOAD are age, female sex, and APOE-ε4 carriership (Liu et al., 2013; O’Neal, 2024; Riedel et al., 2016). Therapeutic development requires disease models that reflect the biological risk factors as well as disease-like phenotypes. As noted in two recent reviews, the majority of mouse models used are the 5xFAD and the 3xTg, which represent the disease process well in terms of amyloid deposition and atrophy, but are suboptimal in terms of their representativeness of human disease (Balu et al., 2019; Jullienne et al., 2022). Despite the fact that these mice replicate the disease process, the individual genetic mutations required are rare and not observed in combination in the human population (Granzotto et al., 2024; Tanzi, 2012). Single mutations result in an early age of disease onset (translating to approximately 30 years of age in the human, mimicking early-onset AD, rather than late-onset), an inconsistent disease profile in male mice, and mouse APOE rather than human APOE, which does not confer the same disease mechanisms (Balu et al., 2019; Granzotto et al., 2024; Jullienne et al., 2022; Tai et al., 2017). Given that the APOE-ε4 allele is the strongest genetic risk factor for LOAD, rodent models that include humanized APOE (hAPOE) are essential for translationally relevant therapeutic development.

To date there are multiple targeted replacement, knock-out, and knock-in models for APOE mice. These mice do recapitulate some key aspects of disease, including cognitive impairment (McLean et al., 2022; Sepulveda et al., 2022). To address the paucity of AD relevant pathology, multiple APOE models have been developed that include other non-dominant mutations, including *Trem2* (Kotredes et al., 2021; Shang et al., 2020), humanized *NOS* (Badea et al., 2019a,b), or humanized *APP* (Badea et al., 2016). Although beneficial for driving pathology, the isolated role of APOE in AD risk and progression is not clearly identifiable in these models.

To this end, a knock-in mouse model expressing humanized APOE-ε3/ε3 and APOE-ε4/ε4 has been developed by the MODEL-AD Consortium (model-ad.org). Recent work with this model demonstrates that the APOE-ε4/ε4 mice have reduced cerebral metabolism and neurovascular uncoupling (Onos et al., 2024), altered peripheral metabolism (Fleeman et al., 2023b; Foley et al., 2022a), immunologic profiles (Fleeman et al., 2023a,b; Sepulveda et al., 2022), and transcriptomic profiles (Foley et al., 2022a,b), decreased cortical microglial responsiveness (Sepulveda et al., 2024), accelerated endocrinological aging (Wang et al., 2025), as well as subtle cognitive differences (McLean et al., 2022; Sepulveda et al., 2022). Many of these effects are sex-specific (Fleeman et al., 2023a; Foley et al., 2022a,b; Wang et al., 2025), which is consistent with differences in the sex distribution of Alzheimer’s disease. These findings suggest that, in some respects, this mouse model may be a suitable risk model for Alzheimer’s disease.

To date, there are no studies that deeply characterize MRI brain imaging phenotypes of aged humanized APOE-ε3/ε3 and APOE-ε4/ε4 mice developed by MODEL-AD. Past work with mouse models that include humanized APOE in addition to other disease- and inflammation-promoting genetic profiles has demonstrated volumetric decreases associated with the hAPOE-ε4 allele (Badea et al., 2019b; Badea et al., 2022; Shang et al., 2020; Yin et al., 2014) as well as decreased fractional anisotropy in major white matter tracts, the piriform, and hippocampus (Badea et al., 2019b; Shang et al., 2020). However, the extent of these differences is not consistently reported. For example, Badea et al. (2022) and Badea et al. (2019a) reported no decreases in hippocampal volume, while Yin et al. (2014) reported lower volume for hAPOE-ε4 mice (Badea et al., 2019b; Badea et al., 2022; Yin et al., 2014). These contradictory findings are similar to those reported for hAPOE-ε4 FAD models (Balu et al., 2019) as well as 3xTg and 5xFAD models (Jullienne et al., 2022). Important confounding effects should be noted for these studies, including other genetic mutations, inconsistent control groups, and varied methods for adjusting regional volumes for total brain volume. Collectively, these factors limit the interpretability of hAPOE-related effects in these mouse models as well as the translational potential for therapeutic development.

The purpose of the present study was to investigate the effects of age, sex, and hAPOE genotype on total brain and localized brain volumes in the MODEL-AD humanized APOE mouse model. As a candidate AD risk model, we hypothesized that humanized APOE-ε4/ε4 mice would exhibit evidence of atrophy and that this effect would be exacerbated in female mice compared to males.

## 2 Materials and methods

### 2.1 Animals

All animal studies were performed following National Institutes of Health guidelines on the use of laboratory animals and all protocols were approved by the University of Arizona Institutional Animal Care and Use Committee. Homozygous humanized APOE-ε4 targeted replacement mice were obtained from Jackson Laboratory (#027894). Humanized APOE-ε3 target replacement heterozygous mice were obtained from Jackson Laboratory (#029018) and bred to get homozygous APOE-ε3 mice. Mice were housed in sterile conditions on 14-hour light, 10-hour dark cycles and provided *ad libitum* access to food and water.

### 2.2 Image acquisition

Mice were sacrificed at prespecified ages (6, 9, 15, and 23–25 months) for *ex vivo* MRI. All animals underwent identical transcardial perfusion fixation irrespective of age group, genotype, or sex: PBS perfusion followed by 4% PFA perfusion, with 48-hour post-fixation storage followed by transfer to 0.01% sodium azide for long-term storage. Brains were retained within the skull prior to imaging, consistent with best-practice recommendations for preserving brain morphometry in *ex vivo* MRI studies (De Guzman et al., 2016). Prior to MRI, fixed skulls were placed in vacuum-desiccated 10 mL syringes filled with Fluorinert (FC-3283) for imaging. High resolution structural images were acquired with a T2-weighted rapid acquisition with relaxation enhancement (RARE) sequence (TE: 30 ms; TR: 1800 ms; flip angle 180°; RARE factor: 8; number of averages: 2; FOV: 2.4 * 1.44 * 0.96; acquisition matrix 320 * 192 * 120; reconstructed voxel size 75 μm; total acquisition time: 3 h and 4 min).

Post-perfusion storage duration prior to MRI was recorded for all animals. Storage duration varied across cohorts as a function of the cross-project acquisition and imaging availability: male animals in the 6-, 9-, and 15-month age groups were stored for a mean of 564, 583, and 582 days respectively (SD: 10–19 days), while female animals of the same age groups were stored for a mean of 74, 56, and 78 days (SD: 20–23 days). Animals in the 24-month cohort were stored for approximately 146–158 days regardless of sex (SD: 72–88 days). The systematic difference in storage duration between sexes at younger age points is a documented characteristic of the dataset and is addressed analytically in Section “2.3 Statistical analyses” and interpreted in Section “4.2 Total brain volume.”

#### 2.2.1 Imaging minimal pre-processing

All preprocessing on and analyses were conducted on the high performance computing cluster at the University of Arizona. All data were preprocessed using a custom pipeline written in Bash and Python. A custom Apptainer container was created with necessary softwares for version control and was used unless otherwise noted. This included ANTs (Tustison et al., 2021) (v. 2.4.4), FSL (Jenkinson et al., 2012) (v. 6.0.6.4), MRtrix (Tournier et al., 2019) (v. 3.0.4), minc-toolkit-extras^1^ (commit 544485d), optimized_antsMultivariateTemplateConstruction^2^ (commit ca4ba15) and Python (v 3.10.12).

#### 2.2.2 Anatomical preprocessing

T2-weighted images underwent initial automated quality control (Kalantari et al., 2024). Outlier images were examined visually and images with significant non-uninform intensities (*n* = 4) were excluded. All included images were reoriented to RAS ordering and intensity-normalized using percentile-based histogram rescaling. Foreground estimation was performed via Otsu thresholding, followed by centering, padding, and cropping to a constrained field of view. Bias field correction was applied using N4BiasFieldCorrection (Tustison et al., 2010), with masks refined through iterative affine and nonlinear registration to the DSURQE (Dorr et al., 2008; Richards et al., 2011; Steadman et al., 2014; Ullmann et al., 2013) mouse brain template^3^ resampled to 0.075 mm isotropic voxel size. Non-local means denoising (Manjón et al., 2010) preceded final bias correction and intensity rescaling. Brain masks were generated by inverse warping of the template mask, yielding a final preprocessed T2w image and corresponding brain mask.

#### 2.2.3 Deformation based morphometry

An initial minimum deformation template was built using optimized_antsMultivariateTemplateConstruction. A total of 96 mice were used to build the template and included 4 mice from each age (6, 9, 15, 24 months), genotype (hAPOE-ε3/3, hAPOE-ε3/4, hAPOE-ε4/4), and sex (male, female) combination. The process began by building an initial template using six iterations of rigid-only transformations to align the images. The resulting average from the final rigid iteration was then used as the target for the next stage, which built a new template using six iterations of similarity transformations. This procedure was sequentially repeated for six iterations of affine transformations and finally for six iterations of non-linear Symmetric Normalization (SyN) transformations. At each iteration of every stage, all subjects were registered to the target template; a new template was then generated by averaging the warped images, sharpening the result, and applying the scaled, inverted average of the transformations for use in the next iteration. A final consensus mask was also generated, computed as the mean of the template-space transformed brain masks and thresholded to include voxels in at least 50% of the masks.

After this process, all of the brains (*n* = 159) were independently re-registered to the minimum deformation template using a single iteration step identical to the template building process, which included both linear (rigid, affine) and nonlinear (SyN) registration components. Deformation based morphometry was implemented through optimized_antsMultivariateTemplateConstruction to produce absolute and relative log-transformed Jacobian determinants of the deformation fields transforming each individual mouse brain into the study-specific template space. Log-transformed Jacobians describe the amount of deformation to warp the template voxel to the individual mouse such that values > 0 indicate larger individual volumes and values < 0 indicate larger template volumes. The relative Jacobians, which exclude the affine and residual affine components of the transformations, were used for all analyses and intrinsically control for total brain size. All images were smoothed with a 4 voxel full width at half maximum kernel prior to analyses.

### 2.3 Statistical analyses

Primary analyses were conducted in template voxel space using Convoxel and ModelArray (Zhao et al., 2023; Zhao et al., 2025) (v. 0.1.5). ModelArray runs in R (R Core Team, 2024) (v. 4.1.2). As a cross-sectional design with predefined age timepoints, reported age effects reflect differences between animals of different ages at the time of imaging rather than longitudinal change within individuals.

Voxelwise linear additive models were fit using ModelArray to examine the independent effects of age, sex, and genotype with sum to zero contrast coding for interpretable coefficients. Specifically, age was Z-scored (mean = 13.4 ± 5.94 months), sex compared males - females, and genotypic effects compared hAPOE-ε3/4 - hAPOE-ε4/4 and then hAPOE-ε3/3 - hAPOE-ε4 carriers. Given sample sizes of *n* = 3–10 per age × sex × genotype combination, the design was insufficiently powered for two- and three-way interactions and were therefore excluded. Voxelwise statistical significance was set at a two-sided pFD < 0.05. Statistically significant voxels were separated into positive and negative directionality (depending on the contrast; i.e., positive: male > female, negative: female > male), localized using the Dorr mouse brain atlas which had been transformed to study-specific template space and summarized as the percent of the region occupied by significant voxels (regional specificity) and the percent of the significant voxels represented by the region (pattern representation). Total brain volumes were additionally analyzed by inverse warping the template space mask to the individual mouse brains, summing the voxels and fitting a linear model with the same covariates as the voxelwise models.

To assess potential confounding by post-perfusion storage duration, three sensitivity analyses were conducted. First, a storage-adjusted model was fit for total brain volume and the voxelwise analyses in the full cohort by adding storage duration as an additional covariate to the primary model. Generalized variance inflation factors (GVIFs) were computed for all terms in the total brain volume model using the *car* package in R (Fox and Weisberg, 2019). Because the design matrix is identical across all covariate-complete voxels, these VIF values apply to voxelwise analyses as well, though voxels with missing data may have locally different collinearity properties. Second, we repeated the analyses with storage duration as a covariate while restricting the data to the 24-month cohort, which had more balanced post-fixation storage times between males and females (males: 146 days ± 88; females: 158 days ± 72). Third, to assess whether storage differentially impact specific tissue classes, total tissue type volumes (gray, white, CSF) were estimated using the Dorr atlas labels in native space and analyzed using the same design matrix, including storage duration.

To assess spatial reproducibility across model specifications, Spearman correlations between t-statistic and beta-coefficient maps were computed across all voxels for each contrast of interest. *Post-hoc*, the minimum detectable effect size for the voxelwise hAPOE contrasts was estimated at each voxel from the residual standard error and degrees of freedom at 80% power and α = 0.05 (two-sided), using the formula MDE = (t_α/2 + t_β) × SE, where t_α/2 and t_β are the critical values at the voxel-specific degrees of freedom. Median MDE values are reported in log-Jacobian units and converted to approximate percentage local volume differences as exp(MDE) - 1.

**FIGURE 1:**
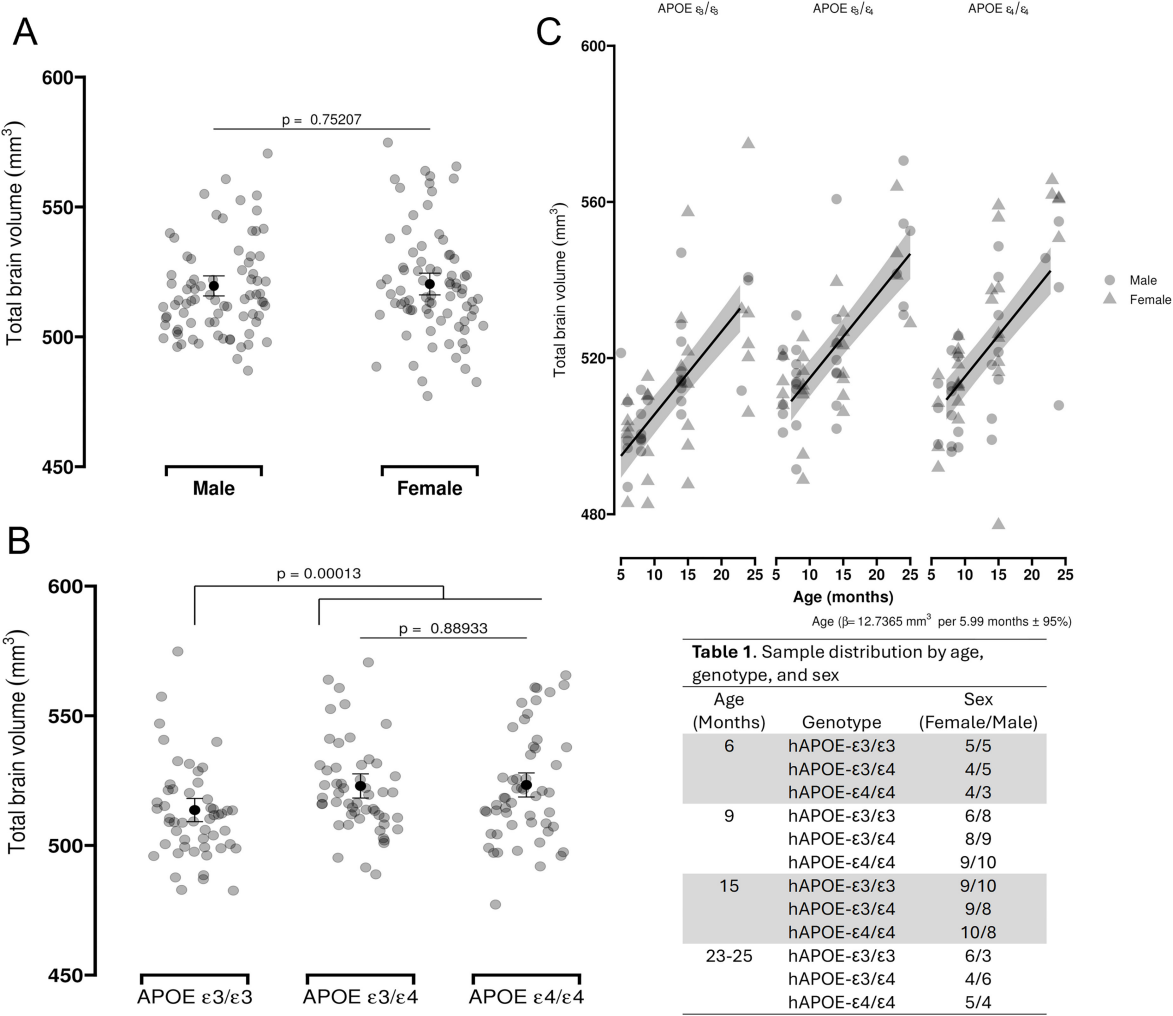
Sex, genotype, and age effects on total brain volume. **(A)** There were no statistically significant differences between male and female mice. **(B)** hAPOE-ε4 carriers exhibited greater volume than hAPOE non-carriers. **(C)** A statistically significant increase in brain volume as observed with increasing age. Group mean and error bars are model-derived marginal means + 95% CI **(A,B)**. Line and ribbon **(C)** represent model fit + 95% CI.

**FIGURE 2:**
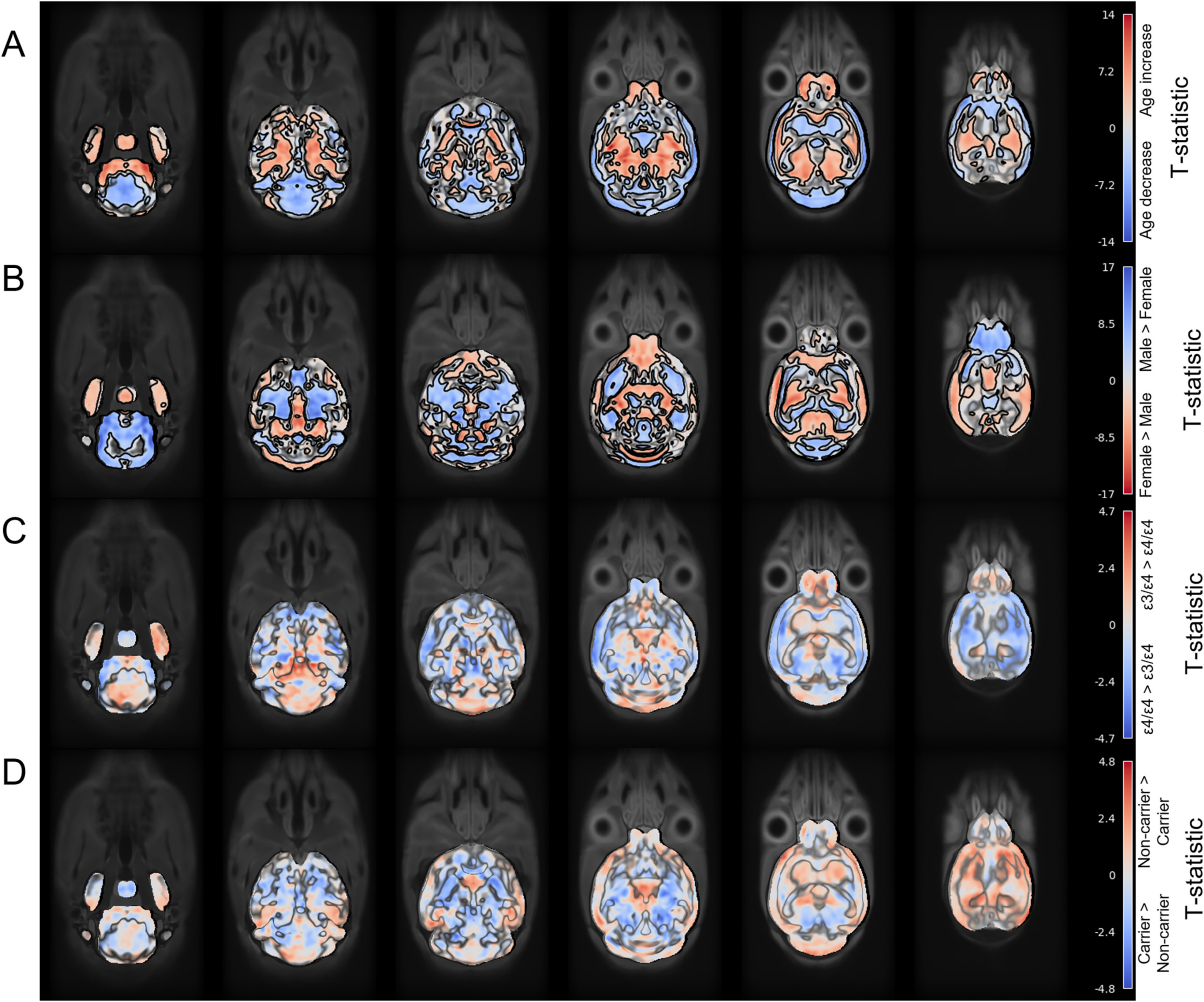
Voxelwise t-statistic maps for main effects of age, sex, hAPOE-ε4 dose, and hAPOE-ε4 carriership. Statistically significant findings (pFDR < 0.05) are outlined and opaque for age **(A)** and sex **(B).** Genotypic effects **(C,D)** are shown as unthresholded t-statistic maps as neither hAPOE contrast survived FDR multiple comparisons correction.

**FIGURE 3:**
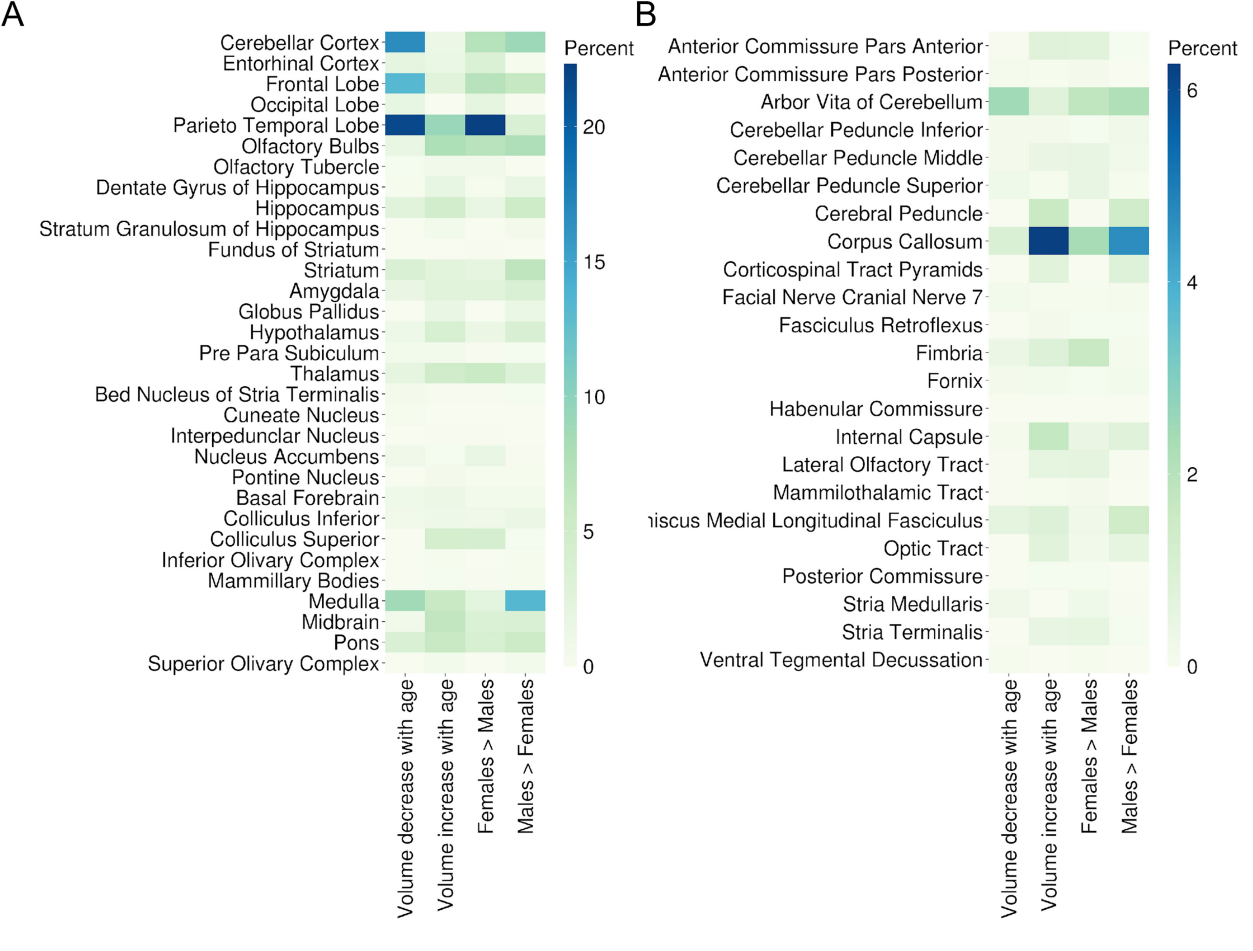
Pattern representation in gray **(A)** and white **(B)** matter. For each effect (age decrease, increase; sex differences), the heatmap indicates the proportion of statistically significant voxels within each region (i.e., > 20% of the voxels in demonstrating an age-related decrease in volume were in the parieto-temporal lobe).

**FIGURE 4:**
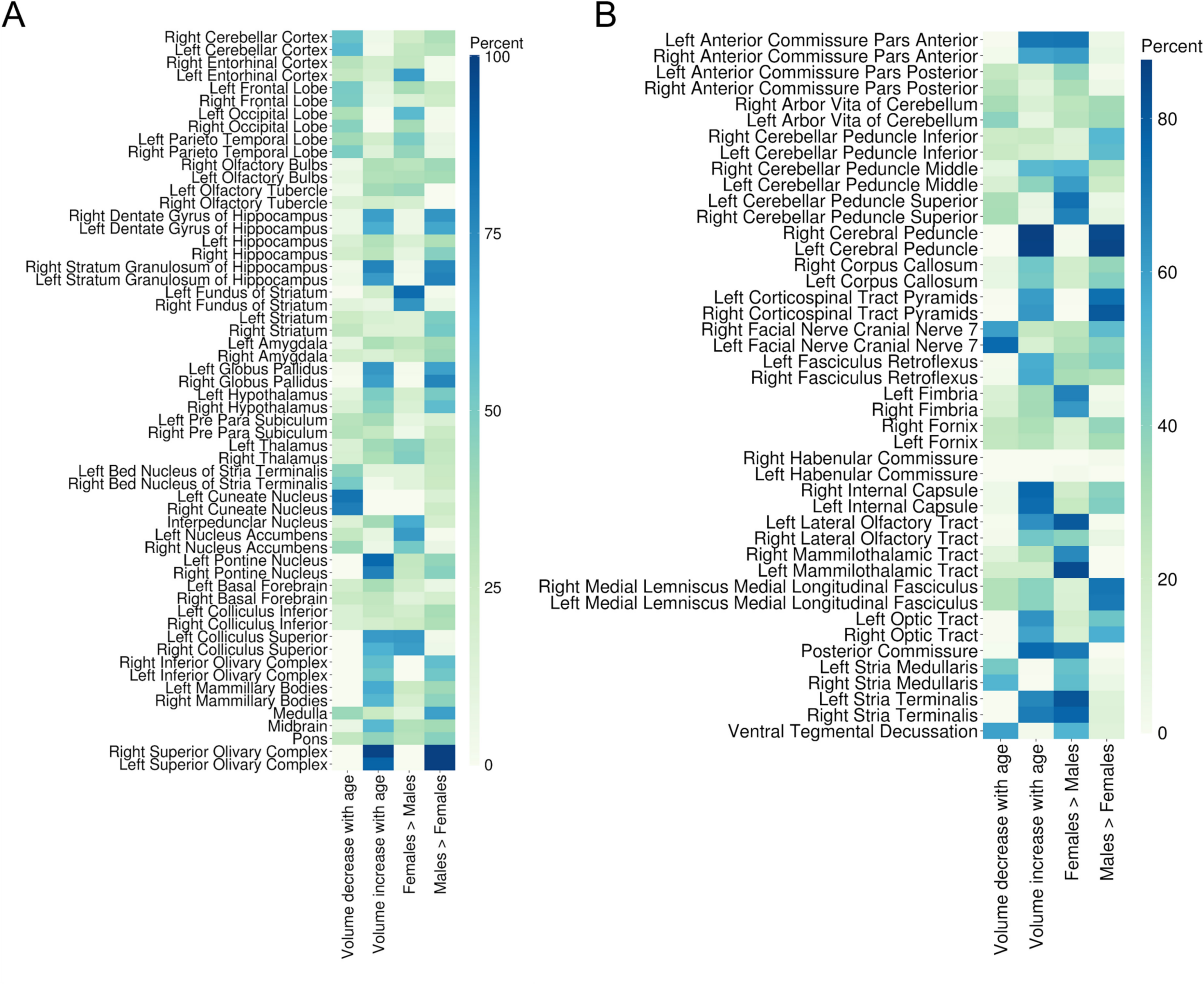
Regional specificity of effects for gray **(A)** and white **(B)** matter. For each effect (age decrease, increase; sex differences), the heatmap indicates the proportion of the regions voxels that are statistically significant (i.e., > 80% of the voxels in the bilateral cerebral peduncles were larger in males compared to females.

Plots were generated using a combination of software including *ggprism* (Dawson, 2024; Figure 1), *nilearn* (Chamma et al., 2025; Figure 2), and *tidyplots* (Engler, 2025; Figures 3, 4).

## 3 Results

### 3.1 Cohort characteristics

A total of 158 mice were included in the present analyses. Distributions by age, sex, and genotype are presented in Figure 1. In cross-sectional analyses, total brain volumes were significantly greater in older animals across the 6–25 month age range (β = 12.74 mm^3^ per SD of age, SE = 1.15, t(152) = 11.04, 95% CI [10.46, 15.02], *p* < 0.0001; equivalent to approximately 2.12 mm^3^ per month of age, where 1 SD = 5.94 months). After controlling for all other parameters, hAPOE-ε4 non-carriers (ε3/3) had lower total brain volumes than hAPOE-ε4 carriers (β = −9.48 mm^3^, SE = 2.41, t(152) = −3.93, 95% CI [−14.25, -4.72], *p* = 0.0001). No statistically significant differences were observed in total brain volumes between hAPOE-ε3/4 and hAPOE-ε4/4 mice (β = −0.38 mm^3^, SE = 2.76, t(152) = −0.14, 95% CI [−5.84, 5.07], *p* = 0.889) or between males and females (β = −0.72 mm^3^, SE = 2.28, t(152) = −0.32, 95% CI [−5.23, 3.79], *p* = 0.752). Total brain volume analyses are summarized in Figure 1.

To assess the effect of storage on total brain volume estimates, two storage-adjusted models were fit as described in Section “2.3 Statistical analyses.” In the full cohort, variance inflation factor analysis confirmed that age (GVIF = 1.25) and hAPOE genotype (GVIF = 1.02) were not collinear with storage duration, though sex and storage duration had moderate-to-high collinearity (GVIF_*sex*_ = 4.55; GVIF_*storage*_ = 4.92). This is consistent with systematic storage duration differences between sexes at younger age points. Importantly, both the age effect (β = 13.10, SE = 1.27, 95% CI [10.59, 15.61], *p* < 0.0001) and hAPOE-ε4 carrier effect (β = −9.47, SE = 2.41, 95% CI [−14.25, −4.70], *p* = 0.0001) on total brain volume were preserved in direction, magnitude, and statistical significance. In the 24-month subset of animals, storage duration was not a significant predictor of total brain volume (β = −6.18, SE = 5.60, *p* = 0.282). Smaller brains in the hAPOE ε3/3 mice were observed in this storage-controlled subgroup (β = −20.91 mm^3^, SE = 7.99, *t* = −2.62, *p* = 0.016) and the effect was larger in magnitude than the full-model estimate.

To characterize the contributions of tissue type to the observed age and hAPOE effects on total brain volume age and hAPOE effects, total gray matter (mean intercept: 426.83 mm^3^), white matter (mean intercept: 59.48 mm^3^), and ventricular volume (mean intercept: 7.26 mm^3^) were estimated from the Dorr atlas labels in native space and modeled with the same covariates, including storage duration. Age was a significant predictor of gray matter volume (β = 9.95 mm^3^/SD, SE = 1.05, t(151) = 9.52, 95% CI [7.89, 12.02], *p* < 0.001) and white matter volume (β = 2.18 mm^3^/SD, SE = 0.16, t(151) = 13.68, 95% CI [1.87, 2.50], *p* < 0.001), together accounting for approximately 95% of the total brain volume age coefficient (12.74 mm^3^/SD). The hAPOE ε3/3 vs. ε4-carrier effect was significant in both gray matter (β = −7.67 mm^3^, SE = 1.99, *p* < 0.001) and white matter (β = −1.24 mm^3^, SE = 0.30, *p* < 0.001), together accounting for 94% of the total brain volume hAPOE carrier effect (−9.48 mm^3^). Storage duration was not a significant predictor of gray matter (*p* = 0.462) or white matter (*p* = 0.063) volume. Age was a predictor of ventricular volume in the unadjusted model (β = 0.11 mm^3^/SD, SE = 0.03, *p* = 0.003), which was attenuated but still significant when storage duration was included (β = 0.09, *p* = 0.02). Neither hAPOE genotype, sex, nor storage duration was a significant predictor of ventricular volume. These results confirm that the age and hAPOE effects on total brain volume primarily reflect distributed differences across gray and white matter compartments.

### 3.2 Older age is associated with cortical volume differences and subcortical preservation in cross-sectional analyses

Age-specific effects are presented in Figure 2A. Greater volumes in older animals were primarily observed in subcortical regions −- including corpus callosum, thalami, hippocampi, superior colliculi, hypothalami, and amydalae −- as well as a portion of the bilateral parieto-temporal lobe accounting for approximately 40% of the total number of significant voxels. Notably while this distribution of age-positive voxels was observed across multiple regions, the regional specificity (proportion of a region with significant voxels) was relatively smaller. Smaller gray matter nuclei (e.g., superior olivary complex, pontine nuclei) as well as smaller white matter pathways (anterior commissure, stria terminalis) and gray matter substructures (dentate and stratum granulosum of the hippocampus) demonstrated age-related regional specificity, with significant voxels in > 60% of those individual regions. Figure 3 (age increase) and Figure 4 (age increase) show the pattern representation and regional specificity for subcortical age-positive voxels, respectively. The pontine nuclei and superior olivary complexes account for the highest regional specificity.

The pattern of lower volume in older animals was substantially represented by the bilateral parieto-temporal cortex (21.7% of significant voxels), cerebellar cortex (16.66% of significant voxels), and frontal lobes (13.39% of significant voxels). To a lesser extent, other regions, including the bilateral striata, hippocampi, entorhinal cortex, amygdala, and thalami were also represented in the overall pattern. Notably, these cortical and cerebellar cortical regions, as well as the occipital lobe, also demonstrated high regional specificity, with up to 50% of the regional voxels demonstrating age-related decrease. Figure 3 (age decrease) and Figure 4 (age decrease) show the pattern representation and regional specificity for cortical age-negative voxels, respectively.

The spatial pattern of age effects was highly stable across sensitivity analyses. The age t-statistic map from the primary model and the storage-adjusted full-cohort model showed a Spearman spatial correlation of *r* = 0.94 and nearly identical effect magnitudes (mean Δβ = 0.0003 log-Jacobian units, ∼0.03%), confirming that the regional pattern described above is not substantially altered by inclusion of storage duration as a covariate.

### 3.3 Cortical and subcortical structures exhibit sexual dimorphism

Sex-specific effects from the primary model are presented in Figure 2B. The regional pattern of male positive effects included the bilateral cerebellar cortices, olfactory bulbs, striata, frontal cortex, hippocampi, and corpus callosum. High regional specificity was observed in the bilateral olivary complexes, white matter pathways (cerebral peduncles, corticospinal tract pyramids, medial longitudinal fasciculus) as well as subcortical structures (globus pallidus, bilateral stratum granulosa and dentate gyri of the hippocampus). The bilateral olivary complexes and cerebral peduncles demonstrated the highest regional specificity for male-biased effects (Figures 3–4).

In contrast, females exhibited greater cortex-dominant regional patterns, with bilateral parieto-temporal, frontal, cerebellar, and entorhinal cortices all substantially contributing to the overall female-specific pattern. White matter tracts (mammilothalamic, stria terminalis, olfactory, cerebellar peduncle superior, anterior commissure), bilateral subependymale zones rhinocele, and the entorhinal cortex further contributed to this female-specific pattern. Full regional pattern representation and specificity for female-biased effects are shown in Figures 3–4.

The sex-specific regional patterns reported above should be interpreted in light of the variable storage durations across cohorts. Comparison of the sex beta-coefficient maps between the primary and storage-adjusted model revealed negligible mean differences in effect magnitude (mean Δβ = −0.003 log-Jacobian units, ∼0.3%), but low spatial consistency (Spearman *r* = 0.20 on beta maps; *r* = 0.24 on t-statistic maps), indicating that the regional distribution of sex effects is not spatially reproducible in the storage-balanced subgroup.

### 3.4 APOE genotype does not drive localized volumetric differences

No voxelwise differences survived multiple comparisons correction for any of the hAPOE genotype analyses, after accounting for age and sex effects (Figures 2C–D). *Post-hoc* analysis indicated that the analyses were sufficiently powered to detect local hAPOE-related volume differences of approximately 3.0% or larger (median minimum detectable effect size = 0.030 log-Jacobian units at 80% power, α = 0.05, across 1,227,270 voxels). The storage-adjusted model yielded an essentially identical median minimum detectable effect (0.030 log-Jacobian units), confirming that storage duration does not affect the precision of the hAPOE genotype estimates at the voxelwise level. To further assess robustness to the storage duration confound, the voxelwise analysis was additionally conducted in the 24-month cohort with storage duration included as a covariate. No voxels survived FDR correction for either hAPOE contrast in this subgroup. The spatial correlation between hAPOE t-statistic maps from the full and 24-month models was moderate (Spearman *r* = 0.43), indicating partial spatial consistency of the null result across model specifications. Given the substantially reduced degrees of freedom in the 24-month subgroup (median MDE ∼7.9%), this analysis serves as a consistency check rather than an independent replication. The lack of statistically significant voxels across analyses and moderate spatial agreement with the full model (*r* = 0.43) confirm that storage duration does not account for the null finding, while the reduced power of this subgroup means that small effects below ∼7.9% local volume difference cannot be excluded in 24-month animals specifically.

## 4 Discussion

### 4.1 Key findings

The present study evaluated whether a humanized APOE mouse model recapitulates MRI-derived volumetric phenotypes associated with aging and Alzheimer’s disease risk. This mouse model was developed as a candidate for translationally relevant research in Alzheimer’s disease addressing the hypothesis that hAPOE genotype would be a significant driver of Alzheimer’s-like morphological phenotypes. Contrary to this hypothesis, there were no voxel-level differences between hAPOE-ε3/ε3 and hAPOE-ε4 mice after controlling for age, sex, and total brain volume. These findings were robust to storage duration prior to imaging in both the full cohort as well as in the storage-controlled 24-month subgroup. Critically, these analyses were sufficiently powered to detect voxel level differences as small as 3%, which is consistent with the smallest effects observed in other work.

Additional findings reported herein indicate that: A) both age and hAPOE-ε4 carriership significantly increase total brain volumes in this model, with the hAPOE-ε4 carrier effect larger in the 24-month subgroup (∼21 mm^3^) than in the full model (∼10 mm^3^); B) localized age-related brain morphometry effects are independent of the global age-related increase, with widespread decreases particularly in cortical volume; and C) sexually dimorphic patterns of local volume differences exist, though these findings require interpretative caution due to storage duration differences across sex and age cohorts. Collectively, these findings indicate that this model does not reproduce the atrophy phenotype observed in human brain aging or Alzheimer’s disease. Instead, the observed pattern is more consistent with what we term “vulnerable aging,” here defined as aging in the presence of genetic risk for neurodegeneration in the absence of histopathological hallmarks, consistent with the accelerated endocrine and bioenergetic aging previously reported in this model (Fleeman et al., 2023b; Foley et al., 2022a; Onos et al., 2024; Wang et al., 2025).

### 4.2 Total brain volume

The present findings indicate that brain volumes in this mouse model continue to increase over the lifespan. This is consistent with findings in different mouse models, including the background C57BL/6 strain, where rapid brain growth is observed over the first few months of life followed by a much slower increase or plateauing of brain volumes over the course of aging (Jullienne et al., 2022). These findings are dissimilar to findings in human aging, where the brain reaches peak volume within the first two decades of life, followed by plateauing or decline in volume over the next 50–70 years (Bethlehem et al., 2022). Normal whole brain volume may decline at rates of up to < 0.5% per year, depending on decade of life (Fujita et al., 2023) and requires between 30 and 35 years for volume to decrease by two standard deviations from its value at age 40 during healthy aging (Vinke et al., 2018). In AD, the rate of decline is accelerated (Bethlehem et al., 2022). The opposing volumetric trajectories between this model and human aging does not limit utility. The hAPOE-relevant effects on cerebral metabolism, neurovascular coupling, neuroinflammation, and transcription that are well-documented in this model (Fleeman et al., 2023a,b; Foley et al., 2022a,b; McLean et al., 2022; Onos et al., 2024; Sepulveda et al., 2022; Sepulveda et al., 2024; Wang et al., 2025) are mechanistically upstream of MRI detectable macrostructure. Accordingly, the most appropriate use of this model is not as a surrogate for late-stage human brain aging or neurodegeneration, but as a controlled genetic risk platform for evaluating interventions at the earliest phases of the hAPOE-related pathological cascade, where treatment effects in humans are also most likely to be meaningful.

Independent of the observed aging effects, a small, statistically significant amount of the variability in total brain volume was explained by genotypic variation, with hAPOE-ε4 carriers having greater brain volumes than hAPOE-ε4 non-carriers. The observed magnitude (9.48 mm^3^) is small and may not represent a translationally relevant magnitude of difference. For reference, this magnitude of difference is approximately the same size as the left thalamus distributed over the entire mouse brain. While APOE-ε4 mediated whole brain atrophy is evident in Alzheimer’s disease, recent studies using large cohorts reflecting presumably healthy aging have revealed subtle or absent APOE genotypic effects on total gray matter and total brain volume (Heise et al., 2024; Lyall et al., 2020; Stein et al., 2012), which is broadly consistent with the findings here. Further, after accounting for age and hAPOE genotype, no sex differences were evident in total brain volumes. Sex differences in total brain volume are robustly reported across the human literature, yet absent in the present analyses (Bethlehem et al., 2022; Ruigrok et al., 2014; Sanchis-Segura et al., 2019; Sang et al., 2021). MRI findings from the background strain (C57BL6) show the same lack of total brain volume differences as observed here (Guma et al., 2024), suggesting that this is not a by-product of the inclusion of humanized hAPOE but rather a mouse-specific phenotype.

These cross-sectional total brain volume effects were robust to the documented effects of long-term post-fixation storage, including general tissue shrinkage and ventricular volume changes (De Guzman et al., 2016). Sensitivity analyses including storage duration as a covariate demonstrated comparable magnitude and direction for both age and hAPOE effects. In the 24-month cohort, where storage durations were comparable between sexes (males: 146 ± 88 days; females: 158 ± 72 days), storage duration was not a significant predictor of total brain volume (β = −6.18, SE = 5.60, *p* = 0.282), while the hAPOE ε3/3 vs. ε4-carrier contrast remained significant and was larger in magnitude than the full-model estimate (β = −20.91 mm^3^, SE = 7.99, *p* = 0.016). A storage-artifact explanation would predict attenuation of the hAPOE effect when storage is controlled; the observed amplification is directionally inconsistent with this prediction and supports the interpretation of the hAPOE-ε4 carrier total brain volume effect as a biological signal.

To assess whether ventricular volume specifically contributed to these total brain volume findings, ventricular volumes were extracted using the Dorr atlas (cerebral aqueduct, bilateral lateral ventricles, third and fourth ventricles) and modeled with the same covariates. Mean total ventricular volume was 7.23 mm^3^ (approximately 1.4% of mean total brain volume), and the age coefficient for ventricular volume was 0.09 mm^3^ per SD of age, accounting for less than 1% of the corresponding total brain volume age coefficient (12.74 mm^3^/SD). Neither hAPOE genotype, sex, nor storage duration was a significant predictor of ventricular volume. By contrast, effect directionality was consistent for age and hAPOE-ε4 carriership in both total gray and white matter volumes. Collectively, these results confirm that the total brain volume age and hAPOE effects reflect distributed parenchymal changes rather than fixation artifact or ventricular expansion, and suggest that this model more reliably captures whole-brain aging processes than hAPOE isoform-specific structural phenotypes.

### 4.3 Aging results in decreased cortical and preserved or increased subcortical volume

While overall increases in brain volume occurred over the aging process, local age-related differences in relative volume estimated from the deformation fields and therefore independent of total brain volume exhibited a spatially heterogeneous pattern. Because the relative Jacobian intrinsically controls for total brain size, the regional decreases in cortical volume reported here reflect local tissue differences independent of observed total brain volume increase. Specifically, the cortex exhibited decreased volume while subcortical regions exhibited more frequently preserved or increased volumes relative to total brain volume. This age-related pattern is evident in both human (Bethlehem et al., 2022; Williams et al., 2021) and mouse (Clifford et al., 2023) literature. Prior work in a related but different mouse model suggests the presence of both metabolic deficits and altered neurovascular coupling in the cortex, which may provide a plausible mechanism for the decreased cortical volume with advancing age (Kotredes et al., 2021; Onos et al., 2024).

Notably, the present mouse model does not display any of the hallmark pathological features of neurodegenerative processes, and thus cortical-subcortical shifts are more consistent non-pathological aging rather than neurodegenerative atrophy. However, the localization and regional specificity of some of the volumetric decline – particularly the parieto-temporal and entorhinal cortices – does reflect early atrophy sites in Alzheimer’s disease (Archetti et al., 2021; Poulakis et al., 2022; Wheatley et al., 2024; Young et al., 2014; Young et al., 2018). This specific decline may therefore reflect a pattern of regional susceptibility consistent with vulnerable aging in the absence of histopathology. The cortical pattern of age-related volume differences in this model which partially overlaps with early AD atrophy sites supports the potential utility as a preclinical risk model, while the absence of overt atrophy and hAPOE genotype effects confirms that additional pathological drivers are required for disease-like structural change.

### 4.4 Cortical and subcortical structures exhibit sexual dimorphism

Interestingly, sex differences were observed in local volumes. Females generally had greater cortical volumes, independent of any age-related decline, than males. These findings are consistent with the pattern of sex differences observed in a comparative study of humans and C57BL/6 mice (Guma et al., 2024), in which consistent sex differences were observed across species with subcortical and deep gray brain structures exhibiting a male bias where cortical regions exhibited a female bias. These findings are generally consistent with other human (Lotze et al., 2019; Ruigrok et al., 2014) and mouse studies (Qiu et al., 2018) of sex differences. These sex-biased regional variations may likely reflect sex hormone exposure and timing in both species (Knickmeyer et al., 2014; Qiu et al., 2018).

### 4.5 APOE genotype does not drive local volume variation

Despite its established role as a major genetic risk factor for Alzheimer’s disease, APOE genotype did not produce detectable voxel-level volumetric differences after controlling for age and sex. This suggests that hAPOE-related effects in this model are either globally distributed, below the spatial sensitivity of volumetric MRI, or require additional pathological or environmental modifiers to manifest structurally. *Post-hoc* power analysis indicated that the voxelwise analyses were sensitive to local volume differences of 3% or greater. This threshold falls within the 2–12% regional effects reported in the closest multi-mutation comparator model incorporating hAPOE plus humanized NOS2 (Badea et al., 2019b), indicating that the null finding is not attributable to insufficient statistical sensitivity. However, effects below 3% voxel volume difference cannot be excluded.

Prior work with this specific mouse model has revealed hAPOE-ε4/ε4 decreases in cerebral metabolism with aging (Onos et al., 2024), alterations in neuronal (Qi et al., 2021) and peripheral metabolism (Fleeman et al., 2023b; Foley et al., 2022a), immunologic profiles (Fleeman et al., 2023a,b; Sepulveda et al., 2022), cortical transcriptomic profiles (Foley et al., 2022a,b), heterogeneous weight trajectories across aging (Vitali et al., 2025), and accelerated endocrine aging (Wang et al., 2025). Mutations (*Trem*2 (Kotredes et al., 2021); *Trem2* + *hAPP* (Kotredes et al., 2024)) in addition to humanized APOE coupled with environmental factors (high fat diet) (Kotredes et al., 2024) revealed reduced cerebral glucose metabolism and neurovascular coupling. In hAPOE-ε4s carrying both *Trem2* and *hAPP* mutations, a high fat diet induced volumetric decreases compared to a normal control diet at multiple age points (Kotredes et al., 2024). Coupled with those findings, the present work supports the interpretation that humanized APOE expression alone is insufficient to induce MRI-detectable macrostructural regional volumetric differences after controlling for age and sex. These data do not address whether hAPOE genotype influences other pathological mechanisms, such as neuroinflammatory, metabolic, or synaptic processes, that may be present below the spatial resolution of voxelwise MRI or in the absence of gross structural atrophy.

Collectively, and in the absence of discernible pathology, these findings point to an aging process characterized by widespread cortical volumetric reductions that are coincident with increased subcortical volumes. These changes are further modified by sex, with female preservation of cortical volumes and male preservation of subcortical volumes, without any specific impact of humanized APOE genotype. These findings partially echo findings from more aggressive AD mouse models that do exhibit pathological hallmarks (amyloid B deposition, tau) (Balu et al., 2019; Jullienne et al., 2022). In those models, age-related cortical thinning and volumetric decrease are greater and more accelerated compared to control mice (Balu et al., 2019; Jullienne et al., 2022). However, the present humanized APOE model, without other aggressive disease-causing mutations, does not reproduce the extent of progressive atrophy observed in aggressive, but translationally constrained, mouse models. Rather, these mice appear to represent a model of vulnerable aging, particularly with hAPOE-ε4/ε4 females demonstrating accelerated aging (Wang et al., 2025), and may serve as a reasonable aging control group when evaluating more aggressive disease models.

### 4.6 Strengths and limitations

This study capitalizes on a number of strengths. Here we pool from a large number of humanized APOE mice across an extensive age range, reflecting an estimated human age range of 30–70. Our imaging pipeline capitalizes on open-source softwares to enable high-throughput processing, robust brain masking and template registration, as well as voxel-wise analyses and ultimately reproducibility within and across research groups. The template brains built from this analytic process can serve as a basis for future target registrations and comparative analyses with other mouse models.

However, several limitations should be acknowledged. First, post-fixation storage times varied across imaged cohorts and the effect of storage time cannot fully be disentangled from sex effects, given that younger male brains were stored considerably longer than either younger females or the oldest mice (young males: 564–583 days; young females: 56–78 days; 24-month cohort: 146–158 days regardless of sex). Prior work documents that up to 3% tissue shrinkage per month may occur during long-term storage in PBS, and that these changes are non-uniform across brain structures (De Guzman et al., 2016). Variance inflation factor analysis confirmed that this collinearity is specific to the sex contrast (GVIF_sex = 4.55) and does not affect the age (GVIF = 1.25) or hAPOE genotype (GVIF = 1.02) estimates. While sex-specific regional patterns reported here are consistent with prior work on mouse brain morphology, the magnitude of the differences reported here should therefore be interpreted with caution, in light of the variable storage duration.

Second, as a cross-sectional study, animals from different age groups were drawn from different birth cohorts and may differ systematically in ways not captured by the model covariates, including litter-of-origin effects, colony-level genetic drift, or microbiome composition. These factors cannot be excluded as contributors to the observed age associations. Longitudinal imaging of individual animals would be required to distinguish within-animal trajectories from cohort-level differences.

Third, the humanized APOE-ε3/3 mouse served as the control group rather than a wild-type (mouse Apoe) mouse. The inclusion of wild-type mice would have permitted assessment of how humanization of APOE alters the mouse brain relative to the murine APOE background. However, murine APOE and human APOE are neither mechanistically nor functionally equivalent (Balu et al., 2019). Direct comparisons between a murine APOE background and humanized APOE isoforms would not be informative about which humanized APOE isoform, ε3 or ε4, more closely recapitulates AD-relevant structural phenotypes. Using the ε3/3 as the control is consistent with isolating humanized APOE isoform-specific effects in preclinical models. Future work comparing humanized APOE mice to a murine APOE background would be valuable for characterizing how humanization itself alters brain structure.

Fourth, two- and three-way interactions between age, sex, and hAPOE genotype were not tested, as the per-cell sample sizes (*n* = 3–10 per age × sex × genotype combination) were insufficient to reliably estimate interaction terms. The presence of sex-specific or age-moderated hAPOE effects cannot therefore be excluded and represents an important direction for future work. Similarly, estrous cycle information was recorded for the younger female mouse cohorts and could be reasonably inferred for the oldest animals. However, inclusion of this information into the models would have (A) further reduced per-cell sample sizes and (B) been largely collinear with age (i.e., 6-month old females were all regularly cycling; no acyclic mice were younger than 15-months), both of which would have made the statistical models underpowered and less stable for the present work. Separate female-specific analyses are underway to disentangle chronological and endocrinological aging effects.

Fifth, the absence of behavioral, molecular, and histological data for the full cohort limits interpretation. Behavioral phenotyping was not conducted across all cohorts; therefore, the relationship between the observed morphometric differences and cognitive or functional outcomes cannot be established from the present data alone. Prior work from this model demonstrates subtle genotype- and sex-related behavioral differences (McLean et al., 2022; Sepulveda et al., 2022), but whether these correspond to the volumetric patterns reported here remains an open question. Similarly, FDG-PET, immunohistochemistry, and other molecular assays were not available for the full cohort. The claim that this model represents non-pathological or vulnerable aging is therefore based on the absence of overt atrophy and on prior characterization studies of this model (Fleeman et al., 2023a,b; Foley et al., 2022a,b; Onos et al., 2024; Sepulveda et al., 2022; Sepulveda et al., 2024; Wang et al., 2025) rather than on histological confirmation of the absence of amyloid or tau pathology in these specific animals. Future work combining MRI with molecular and behavioral phenotyping in the same animals would substantially strengthen the translational interpretation of these findings.

Finally, all animals were housed in sterile environments with a controlled, standard diet. This housing strategy decreases the potential influence of environmental confounders (e.g., bacterial and viral contagions, pollution) on the genetic risk factors at the sacrifice of real world environmental factors that may contribute to disease onset and progression in humans (Granzotto et al., 2024; Reyes-Reyes et al., 2024). Future work investigating the impact of translationally relevant modifiable risk factors, such as diet and environment, would further enhance the translatability of murine models to real-world conditions.

## 5 Conclusion

Volumetric analyses of the humanized APOE mouse model indicate that these mice do not present with an Alzheimer’s disease-like phenotype, despite disease-related risk factors of age, female sex, and hAPOE genotype. These findings suggest that, for these mice, hAPOE-ε4 alone does not confer macrostructural atrophy and likely requires additional risk factors (genetic and/or environmental). These conclusions are specific to voxelwise morphometric MRI within the present cross-sectional design and do not address hAPOE-dependent pathological mechanisms at molecular, cellular, or functional levels. The structural neurodegeneration characteristic of Alzheimer’s disease likely requires additional genetic and/or environmental risk factors beyond hAPOE-ε4 alone. Notably, a significant and unexpected increase in total brain volume increase was observed in hAPOE-ε4 carriers, which was amplified in the 24-month subgroup, suggesting age-dependent hAPOE-related structural signals that may emerge with advancing age or in the context of additional pathological modifiers. These mice may serve as a reasonable aging control group when evaluating more aggressive disease models and as a model of vulnerable aging in preclinical therapeutic development. Analyses are underway with a mouse model that combines humanized APOE genotypes with humanized APP, which may better model the amyloid-generating process and ultimately disease progression.

## Data Availability

The raw data supporting the conclusions of this article will be made available by the authors, without undue reservation.

## Footnotes

1
https://github.com/CoBrALab/minc-toolkit-extras

2
https://github.com/CoBrALab/optimized_antsMultivariateTemplateConstruction

3
https://github.com/IBT-FMI/mouse-brain-templates
